# Nanomaterial-Enhanced Hybrid Disinfection: A Solution to Combat Multidrug-Resistant Bacteria and Antibiotic Resistance Genes in Wastewater

**DOI:** 10.3390/nano14221847

**Published:** 2024-11-19

**Authors:** Tapas Kumar Mandal

**Affiliations:** School of Mechanical Engineering, Yeungnam University, Gyeongsan 38541, Republic of Korea; tpsmndl@yu.ac.kr or tps.mndl@gmail.com

**Keywords:** nanomaterial-based disinfection, antibiotic resistance genes (ARGs), multidrug-resistant bacteria (MDR), photocatalytic wastewater treatment, sonophotocatalysis

## Abstract

This review explores the potential of nanomaterial-enhanced hybrid disinfection methods as effective strategies for addressing the growing challenge of multidrug-resistant (MDR) bacteria and antibiotic resistance genes (ARGs) in wastewater treatment. By integrating hybrid nanocomposites and nanomaterials, natural biocides such as terpenes, and ultrasonication, this approach significantly enhances disinfection efficiency compared to conventional methods. The review highlights the mechanisms through which hybrid nanocomposites and nanomaterials generate reactive oxygen species (ROS) under blue LED irradiation, effectively disrupting MDR bacteria while improving the efficacy of natural biocides through synergistic interactions. Additionally, the review examines critical operational parameters—such as light intensity, catalyst dosage, and ultrasonication power—that optimize treatment outcomes and ensure the reusability of hybrid nanocomposites and other nanomaterials without significant loss of photocatalytic activity. Furthermore, this hybrid method shows promise in degrading ARGs, thereby addressing both microbial and genetic pollution. Overall, this review underscores the need for innovative wastewater treatment solutions that are efficient, sustainable, and scalable, contributing to the global fight against antimicrobial resistance.

## 1. Introduction

The rise of multidrug-resistant bacteria and antibiotic resistance genes in wastewater systems has emerged as a critical global public health and environmental threat. This issue is largely fueled by the excessive and improper use of antibiotics, which promotes the development and spread of resistant bacterial strains and resistance genes, undermining the effectiveness of current antimicrobial treatments. Wastewater systems, especially those linked to hospitals, pharmaceutical facilities, and urban areas, act as major reservoirs and transmission pathways for these resistant elements, facilitating their ongoing dissemination into natural environments and human populations [1]. The presence of MDR bacteria in wastewater is particularly concerning because these pathogens are resistant to multiple antibiotics, rendering conventional treatments ineffective. This resistance complicates the management of infections and increases the likelihood of treatment failures, prolonged illnesses, and higher mortality rates. Furthermore, the wastewater environment, rich in nutrients and organic matter, provides an ideal habitat for the proliferation and persistence of these resistant bacteria [2]. Most multidrug-resistant (MDR) bacteria carry genes that confer resistance to antibiotics, often located on mobile genetic elements (MGEs) like plasmids, transposons, and integrons. These MGEs enable the horizontal transfer of resistance traits between different bacterial species. Horizontal gene transfer (HGT) plays a crucial role in accelerating the spread of resistance, allowing non-pathogenic environmental bacteria to acquire resistance genes and subsequently transfer them to pathogenic bacteria. This process complicates public health efforts to control infectious diseases by facilitating the rapid dissemination of antibiotic resistance [3]. The alarming rate at which ARGs are spreading across various environmental matrices underscores the urgency of developing effective strategies to mitigate this threat [4].

Conventional wastewater treatment processes, which typically include primary, secondary, and sometimes tertiary treatments, are often inadequate in completely removing MDR bacteria and ARGs. Primary treatment processes, such as sedimentation, primarily target the removal of large particles and some suspended solids, while secondary treatments, including biological processes like activated sludge, focus on the degradation of organic matter. However, these processes do not effectively eliminate MDR bacteria or ARGs. In fact, some studies have shown that ARGs can persist and even proliferate during the treatment process, leading to their release into receiving water bodies [5,6]. Recent advancements in treatment technologies, including membrane bioreactors (MBRs) and advanced oxidation processes (AOPs), used alongside traditional disinfection methods like chlorination and UV irradiation, have demonstrated improved efficiency in removing contaminants. However, each approach presents certain drawbacks. For instance, chlorination can result in the formation of hazardous disinfection by-products (DBPs), while UV irradiation may be less effective against certain resistant bacterial strains and can face challenges when water turbidity is high [7,8]. Additionally, these methods often require significant energy inputs and operational costs, making them less feasible for large-scale implementation in resource-constrained settings. Given these challenges, there is a pressing need for innovative and more effective approaches to disinfect wastewater and reduce the prevalence of MDR bacteria and ARGs. Recent advances in nanotechnology offer promising solutions to this problem. Nanomaterial-driven hybrid disinfection approaches, which combine the unique properties of nanomaterials with conventional disinfection techniques, have shown great potential in enhancing the efficiency of wastewater treatment processes.

## 2. Issues with Conventional Wastewater Methods

The increasing prevalence of multidrug-resistant (MDR) bacteria and antibiotic resistance genes (ARGs) in wastewater is a growing concern, posing significant threats to public health and the environment. Despite the critical importance of wastewater treatment plants (WWTPs) as barriers to the spread of these harmful agents, conventional treatment methods often fall short in effectively eliminating MDR bacteria and ARGs. These limitations highlight the need for more advanced and targeted approaches to wastewater treatment. Conventional wastewater treatment typically involves three stages: primary, secondary, and tertiary treatment. Primary treatment focuses on the removal of large solids and particulate matter through physical processes like sedimentation and screening. Secondary treatment primarily involves biological processes, such as activated sludge, trickling filters, or biofilm reactors, to degrade organic matter and reduce biochemical oxygen demand (BOD) [9]. Tertiary treatment, often referred to as advanced treatment, is designed to further polish the effluent by removing nutrients, pathogens, and residual solids through processes like filtration, disinfection, and nutrient removal [10]. While these stages are effective in removing organic pollutants and reducing pathogen loads, they are not specifically designed to address the challenge of MDR bacteria and ARGs. Several factors contribute to the persistence of these contaminants in treated wastewater, which can subsequently be released into the environment, perpetuating the cycle of antimicrobial resistance [11]. Though common disinfection methods including chlorination, ultraviolet (UV) irradiation, ozonation, etc., are effective at inactivating many pathogens, they often fall short when it comes to eliminating MDR bacteria and ARGs [12]. Chlorination, the most widely used disinfection method, relies on the oxidative properties of chlorine to disrupt bacterial cell walls and nucleic acids. However, certain MDR bacteria, particularly those that form biofilms, exhibit enhanced resistance to chlorine, leading to incomplete disinfection [13]. Additionally, the use of chlorine can lead to the formation of disinfection by-products (DBPs), some of which are harmful to human health and the environment [14].

Ultraviolet (UV) irradiation is one of the most widely used disinfection methods. It functions by causing DNA damage in bacteria, inhibiting their ability to replicate and ultimately leading to cell death. However, its efficiency can be reduced in the presence of particulate matter in wastewater, as these particles can shield bacteria from direct UV exposure, limiting its disinfection effectiveness [15]. Furthermore, UV irradiation is less effective against ARGs, which may persist in the effluent even after bacterial cells have been inactivated [16]. Ozone treatment is another widely used disinfection method, utilizing ozone gas to oxidize and inactivate bacteria. It is often more effective than chlorination and UV irradiation, especially against resistant bacterial strains. However, the high operational costs and energy demands of ozonation make it less feasible for widespread application, particularly in low-resource environments [17]. Additionally, ozonation can also lead to the formation of harmful by-products, which necessitates further treatment steps to ensure the safety of the treated effluent [18].

The main concern regarding multidrug-resistant (MDR) bacteria is their capacity to endure and multiply by evading the disinfection conditions typically employed in wastewater treatment plants (WWTPs). The varied microbial communities within these facilities, combined with the selective pressure from antibiotics and other antimicrobial substances present in the wastewater, foster an environment that supports the survival of resistant strains [19]. Additionally, MDR bacteria often possess various resistance mechanisms, such as efflux pumps, enzymatic degradation, and modifications to target sites, which enable them to withstand the effects of antimicrobial agents used in treatment processes [20]. Several studies have reported the presence of MDR bacteria, including strains resistant to last-resort antibiotics like carbapenems and colistin, in treated effluent from WWTPs [21]. These bacteria are often detected at concentrations that pose significant risks to downstream ecosystems and public health. MDR bacteria frequently survive by forming biofilms, which are structured communities of bacteria embedded within a self-produced extracellular matrix. These biofilms create a protective environment that significantly enhances bacterial resistance to various environmental stressors, including disinfecting agents [22]. Conventional disinfection methods, such as chlorination and UV irradiation, often struggle to effectively eliminate multidrug-resistant (MDR) bacteria and antibiotic resistance genes (ARGs) due to factors like biofilm formation and the presence of particulates that shield bacteria from exposure. Additionally, these traditional techniques can produce harmful by-products and may not be effective against all bacterial strains. In contrast, emerging sterilization methods, such as nanomaterial-enhanced photocatalysis and the use of natural biocides, show promise for overcoming these limitations. By generating reactive oxygen species (ROS) and improving charge separation, these novel approaches enhance the overall disinfection efficacy, allowing for more comprehensive control of antibiotic resistance in wastewater environments [23].

**Figure 1:**
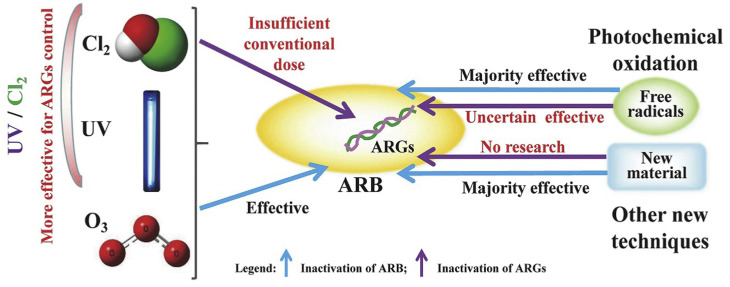
A schematic illustration depicting the limitations of conventional bacterial disinfection methods and the necessity for innovative disinfection strategies. Reprinted with permission from Ref. [23] CC by 4.0.

ARGs, which encode resistance mechanisms that can be transferred between bacteria, represent another critical challenge in wastewater treatment. These genes are often carried on mobile genetic elements (MGEs) such as plasmids, transposons, and integrons, which facilitate horizontal gene transfer (HGT) among bacterial populations [24]. The spread of ARGs through HGT is a major driver of antimicrobial resistance in environmental settings and contributes to the dissemination of resistance traits across diverse bacterial communities. Conventional wastewater treatment processes are not specifically designed to target ARGs, and as a result, these genes can persist through the treatment stages and be released into the environment with the treated effluent [5]. Studies have shown that WWTPs can act as hotspots for the proliferation and dissemination of ARGs, with some treatment processes potentially selecting for resistant bacteria and ARGs under certain conditions [25]. It has also been reported that activated sludge processes, which rely on the growth of microbial communities to degrade organic matter, can inadvertently promote the spread of ARGs through HGT within the microbial consortia. The high microbial density and frequent exchange of genetic material within activated sludge systems provide ideal conditions for the transfer of resistance genes between bacteria, including those that are pathogenic [26]. Likewise, certain treatment processes, such as chlorination, can induce the formation of reactive chlorine species that damage bacterial DNA, potentially leading to the release of free-floating ARGs into the environment. These free ARGs can be taken up by other bacteria through natural transformation, further propagating resistance [27]. Figure 1 illustrates the limitations of traditional bacterial disinfection processes, emphasizing the pressing need for advanced disinfection techniques to effectively combat multidrug-resistant (MDR) bacteria and antibiotic resistance genes (ARGs) in wastewater. Conventional methods, such as chlorination and UV irradiation, often fall short in achieving complete bacterial inactivation due to factors such as the presence of biofilms, particulate matter, and the formation of harmful disinfection by-products. These shortcomings result in insufficient removal of resistant bacteria and the potential for regeneration of viable organisms. In contrast, the figure highlights the advantages of emerging hybrid disinfection strategies, which integrate nanomaterials, natural biocides, and advanced oxidation processes. By leveraging the unique properties of these innovative approaches, such as enhanced reactive oxygen species (ROS) generation and improved charge separation, the techniques promise greater efficacy in targeting both microbial and genetic contaminants, thereby offering a more sustainable and effective solution to the challenges posed by antimicrobial resistance in wastewater treatment systems [23].

## 3. The Role of Different Techniques in Wastewater Treatment

There is a pressing need for innovative and more effective approaches to disinfect wastewater and reduce the prevalence of MDR bacteria and ARGs. Recent advances in nanotechnology offer promising solutions to this problem. Nanomaterial-driven hybrid disinfection approaches, which combine the unique properties of nanomaterials with conventional disinfection techniques, have shown great potential in enhancing the efficiency of wastewater treatment processes [28].

One such approach involves the use of photocatalytic nanomaterials, which have been demonstrated to effectively generate ROS under light irradiation. These ROS, including hydroxyl radicals (·OH) and superoxide anions (O_2_^−^), are highly reactive and can cause significant damage to bacterial cell walls, membranes, and intracellular components, leading to cell death [29]. When combined with other disinfection methods, such as ultrasonication, natural biocides of plant origin like terpenes can act as natural modifiers, and these nanomaterials such as TiO_2_, ZnO, Bi_2_O_3_, and Ag_3_PO_4_ can synergistically enhance the overall disinfection efficiency, offering a comprehensive solution for the removal of MDR bacteria and ARGs from wastewater [30,31,32]. The employment of photocatalytic nanomaterials in wastewater treatment facilitates efficient ROS formation by using light energy, mostly from sunlight or LED sources. This not only reduces the need for chemical disinfectants, which can have adverse environmental effects, but also allows for the potential reuse of treated water, contributing to water conservation efforts [33]. Furthermore, the reusability of nanomaterials over multiple disinfection cycles without significant loss of efficacy makes this approach more sustainable and economically viable [30,34,35]. In addition to their bactericidal effects, photocatalytic nanomaterials can also play a critical role in the degradation of ARGs and MGEs. The reactive oxygen species (ROS) generated during photocatalysis can oxidize nucleic acids, leading to the breakdown of DNA and RNA, thereby reducing the potential for HGT and the spread of resistance [36]. ROS are critical intermediates in photocatalytic processes, playing a pivotal role in the degradation of pollutants and the disinfection of pathogens. Beyond the commonly known hydroxyl radicals (•OH) and superoxide radicals (O_2_^−^), additional ROS like electron–hole (e⁻/h⁺) pairs, hydroperoxide radicals (HO_2_), and persulfate radicals (S_2_O_8_^2^⁻) are essential in enhancing photocatalytic efficiency. In a typical photocatalytic reaction, light absorption generates electron–hole pairs, which initiate redox reactions, leading to the formation of various ROS. These electron–hole pairs are fundamental, as they enable the generation of both oxidative and reductive species, amplifying the disinfection potential. Hydroperoxide radicals are highly reactive and contribute to sustained ROS production, while persulfate radicals, often introduced via persulfate salts, act as potent oxidizing agents, extending ROS action and disinfection efficacy. Intermediate species, which form transiently during these reactions, play crucial roles by enhancing or modulating the activity of primary ROS. By incorporating these additional species into hybrid disinfection systems, we achieve a more comprehensive and potent approach to pathogen inactivation, as each ROS targets different microbial components, from cell walls to intracellular molecules. This broader ROS spectrum thus enhances the overall efficiency and robustness of photocatalytic disinfection, offering an advanced strategy for environmental and public health applications. This dual action of targeting both MDR bacteria and ARGs makes nanomaterial-driven hybrid disinfection methods particularly appealing for addressing the complex challenges posed by AMR in wastewater [37].

The integration of advanced disinfection techniques into existing wastewater treatment infrastructure could significantly enhance the ability to control the spread of AMR. However, the successful implementation of nanomaterial-driven disinfection methods requires careful consideration of various operational parameters, such as light intensity, ultrasonication power, catalyst dosage, and water quality. Optimization of these parameters is crucial in maximizing disinfection efficiency while minimizing potential environmental and health risks associated with the use of nanomaterials [38]. Furthermore, the environmental impact and toxicity of nanomaterials utilized in wastewater treatment must be meticulously assessed to guarantee that their utilization does not pose new environmental risks. Although many studies have demonstrated the effectiveness of nanomaterials in laboratory settings, there is still a need for comprehensive field studies and long-term monitoring to assess their real-world performance and potential impacts on ecosystems and human health [39]. The development and deployment of nanomaterial-driven hybrid disinfection approaches represent a promising frontier in the fight against MDR bacteria and ARGs in wastewater. By leveraging the unique properties of hybrid nanocomposites and other nanomaterials, these methods offer a powerful and sustainable alternative to conventional disinfection techniques [40,41]. This study is motivated by the pressing global challenge posed by the rising levels of multidrug-resistant (MDR) bacteria and antibiotic resistance genes (ARGs) in wastewater systems. Conventional wastewater treatment methods have proven inadequate in effectively reducing these emerging pollutants, leading to increased public health risks and environmental degradation. The limitations of existing disinfection techniques, such as UV irradiation and chlorination, along with high energy costs and the production of harmful by-products, underscore the need for innovative approaches. This research seeks to evaluate the effectiveness of hybrid disinfection systems that incorporate nanomaterial-enhanced photocatalysis and natural biocides in addressing the issues of MDR bacteria and ARGs. The goal is to develop sustainable, efficient, and scalable alternatives that can be safely integrated into wastewater treatment processes, providing more comprehensive and long-lasting solutions to combat antimicrobial resistance.

## 4. Methodology

Nanocomposites are materials that combine nanoscale components, typically with a size range of 1 to 100 nanometers, with a bulk material matrix to create a hybrid structure that exhibits unique physical, chemical, and mechanical properties. The key characteristic of nanocomposites is the dispersion of nanoparticles within the matrix, which could be a polymer, metal, ceramic, or other materials. These nanoparticles, which may be inorganic (e.g., carbon nanotubes, nanoparticles of metals like silver, or clay particles) or organic (e.g., organic dyes, bio-molecules), enhance the overall performance of the composite by improving properties such as strength, thermal stability, electrical conductivity, and chemical reactivity. The main differences between nanocomposites and conventional composites lie in the scale of the reinforcing phase and the resultant properties. While traditional composites have larger reinforcing fibers or particles, nanocomposites exhibit remarkable improvements in material characteristics due to the large surface area and high aspect ratio of the nanoscale reinforcements. The composition of nanocomposites often involves careful selection of the nanoparticle type, size, shape, and dispersion technique to optimize interactions between the matrix and the nanoparticles, leading to the desired enhancement in properties such as barrier performance, UV resistance, and catalytic activity. The interface between the nanoparticles and the matrix is crucial in determining the composite’s final behavior, and this can be further modified by surface functionalization of the nanoparticles to improve compatibility and dispersion within the matrix.

Study Design: This review investigates hybrid disinfection systems in wastewater treatment, with a focus on the integration of photocatalytic nanomaterials, ultrasonication, and natural biocides. The goal is to evaluate their combined efficacy in degrading multidrug-resistant (MDR) bacteria and antibiotic resistance genes (ARGs). Emphasis is placed on the sonophotocatalytic mechanisms and performance, offering insight into their role in enhancing treatment efficiency.

Sources, Search Strategy, and Inclusion Criteria: A systematic literature review was conducted using databases such as Scopus, Web of Science, and Google Scholar, covering studies from 2002 to 2024. The search focused on terms like “hybrid nanocomposites and other nanomaterials”, “sonophotocatalysis”, “blue LED”, “ultrasonication”, “terpenes”, “plant extracts”, “terpene natural products”, and “antibiotic resistance genes (ARGs)” within wastewater treatment contexts. Inclusion criteria were as follows:

I. Studies on photocatalytic nanomaterials (TiO_2_, ZnO, PbS/CaCO_3_, α-Fe_2_O_3_/b-TiO_2_ their hybrid nanocomposites, etc.);
II. Research on hybrid disinfection systems combining sonocatalysis, photocatalysis, and/or biocides;
III. Investigations targeting MDR bacterial inactivation or ARG degradation;
IV. Quantitative data on energy usage or cost-efficiency.

Initial screenings based on titles and abstracts were followed by the removal of duplicates and selection based on inclusion criteria. Detailed data extraction included nanomaterial type, photocatalytic mechanisms, and operational parameters (e.g., ROS generation, light intensity, ultrasonication frequency). Key metrics related to MDR bacterial inactivation, ARG degradation, energy consumption, and cost-effectiveness were recorded.

Data Analysis: The review compared conventional wastewater treatment technologies with modern advanced oxidation processes (AOPs), focusing on hybrid disinfection approaches. The performance of these hybrid systems was analyzed for their ability to eliminate MDR bacteria and degrade ARGs. When relevant, synergy indices quantifying the enhanced effect of combining ultrasonication and photocatalysis were calculated. Energy consumption was evaluated using the standard formula for electrical energy consumption (EEC).

## 5. Advanced Oxidation Processes for Sustainable Disinfection

Advanced oxidation processes (AOPs) are powerful techniques used for the degradation of pollutants and pathogens in water, leveraging highly reactive species such as hydroxyl radicals (•OH) to achieve oxidation. These radicals are non-selective and highly reactive, capable of breaking down complex organic compounds into simpler, often non-toxic, molecules, making AOPs highly effective for environmental remediation. When combined with nanomaterials in hybrid disinfection systems, AOPs are significantly enhanced, as nanomaterials like titanium dioxide (TiO_2_), zinc oxide (ZnO), and silver nanoparticles increase the generation of reactive oxygen species (ROS) under certain conditions, such as UV or visible light exposure. The nanomaterials act as photocatalysts, amplifying the ROS production and enabling rapid disinfection through direct oxidation of pathogens or by disrupting cell membranes via ROS attack. Hybrid disinfection systems that integrate AOPs with nanomaterials provide an effective approach for treating water contaminated with antibiotic-resistant bacteria and other pathogens. The nanoscale properties, such as high surface area and tunable reactivity, allow for greater interaction with contaminants and pathogens, increasing the disinfection efficiency. Furthermore, nanomaterials in AOPs can be modified with functional groups or coatings to target specific pollutants or to improve stability in aqueous environments, which addresses some limitations of conventional AOPs in real-world applications. Thus, nanomaterial-enhanced AOPs represent a promising solution for advanced disinfection, bridging gaps in traditional water treatment technologies and supporting sustainable wastewater management.

Considering the shortcomings of traditional wastewater treatment methods in adequately eliminating multidrug-resistant (MDR) bacteria and antibiotic resistance genes (ARGs), there is an immediate need for advanced treatment technologies capable of tackling these challenges. Innovative strategies, such as hybrid disinfection methods that incorporate nanomaterials alongside physical techniques like ultrasonication and bio-additives, present promising solutions. These approaches leverage the unique properties of nanomaterials in conjunction with conventional treatment processes to enhance the removal efficiency of resistant bacteria and their associated genes [42,43]. The critical question arises: can nanomaterial-enhanced hybrid disinfection systems that integrate photocatalysis, natural biocides, and ultrasonication effectively overcome the limitations of conventional wastewater treatment methods in reducing multidrug-resistant (MDR) bacteria and antibiotic resistance genes (ARGs) while maintaining sustainability, scalability, and energy efficiency? This inquiry seeks to evaluate the innovative potential of combining advanced disinfection techniques to address the inefficiencies and drawbacks inherent in traditional wastewater treatment technologies. The review aims to examine the mechanisms, effectiveness, and practical applicability of these hybrid systems in providing a comprehensive solution to the challenges posed by antimicrobial resistance in environmental settings.

**Figure 2:**
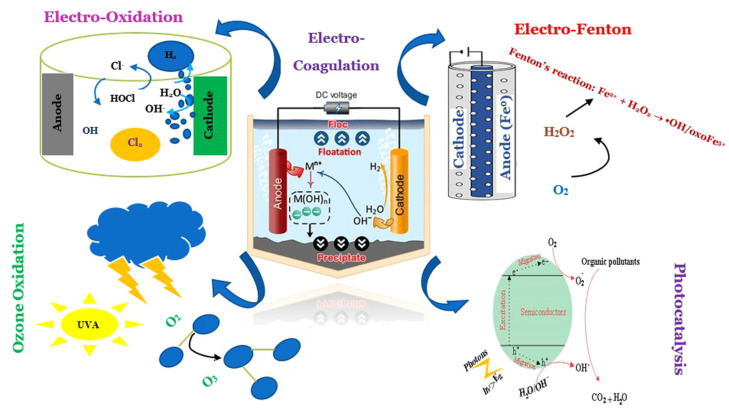
The diagram depicts the primary mechanisms involved in electro-oxidation, Electro-Fenton processes, ozone oxidation, and photocatalysis, all integrated with electrocoagulation for improved elimination of contaminants of emerging concern (CECs). Reprinted with permission from Ref. [44] CC by 4.0.

Figure 2 illustrates the intricate interplay of various advanced oxidation processes (AOPs) employed for the effective removal of contaminants of emerging concern (CECs) in wastewater treatment. The schematic emphasizes how electro-oxidation serves as a foundational technique, utilizing electrical energy to facilitate the oxidation of organic pollutants directly. The incorporation of the Electro-Fenton process enhances this approach by generating hydroxyl radicals (OH), which are highly reactive and capable of degrading a wide range of organic contaminants. Moreover, ozone oxidation contributes another layer of efficiency, leveraging the strong oxidizing properties of ozone to break down resistant compounds. The integration of photocatalysis introduces a synergistic effect, where semiconductor materials, activated by light, facilitate the generation of electron–hole pairs that drive oxidative reactions. Finally, the combination with electrocoagulation aids in the aggregation and precipitation of suspended solids and pollutants, thereby enhancing overall treatment efficacy. This multifaceted approach not only improves the degradation of CECs but also promotes the reduction of by-products, ultimately leading to a more sustainable and efficient wastewater treatment process. The visual representation in Figure 2 underscores the necessity of employing integrated strategies to tackle the challenges posed by emerging contaminants in wastewater systems effectively [44,45,46]. When integrated with existing treatment processes, these nanomaterials can provide a more comprehensive approach to wastewater disinfection, reducing the prevalence of MDR bacteria and ARGs in treated effluent. Additionally, the development of novel disinfection technologies, such as electrochemical disinfection, membrane filtration, and AOPs, can complement conventional methods and provide additional barriers to the spread of antimicrobial resistance. When integrated with existing treatment processes, these nanomaterials can provide a more comprehensive approach to wastewater disinfection. For example, titanium dioxide (TiO_2_) nanoparticles have been shown to enhance the efficacy of UV irradiation by generating additional ROS, leading to improved inactivation of MDR bacteria and degradation of ARGs [47]. Similarly, silver nanoparticles (AgNPs) have been found to possess strong antimicrobial properties, making them effective in reducing the microbial load in wastewater, including resistant strains [48]. Electrochemical disinfection is another advanced method that has shown promise in addressing the challenges of conventional disinfection. This process involves the generation of reactive chlorine species (RCS) and ROS through the application of an electric current, which can inactivate bacteria and degrade ARGs more effectively than traditional methods [49,50]. Electrochemical disinfection can be integrated with nanomaterials to further enhance its efficacy, providing a more sustainable and energy-efficient solution for wastewater treatment [51]. Membrane filtration is another advanced technology that offers significant advantages over conventional disinfection methods. Membranes with nanoscale pores can physically remove bacteria and ARGs from wastewater, providing a barrier to the release of these contaminants into the environment [52]. Furthermore, the integration of nanomaterials into membrane systems can enhance their antimicrobial properties, reducing the risk of membrane fouling and improving overall treatment efficiency [53]. AOPs, which involve the generation of highly reactive hydroxyl radicals (•OH), have also been explored as a means of enhancing wastewater disinfection. These radicals are capable of degrading a wide range of organic contaminants, including emerging pollutants and ARGs, making AOPs a versatile and effective treatment option [54]. The use of nanomaterials in AOPs, such as iron oxide nanoparticles, can further enhance the generation of hydroxyl radicals, improving the overall disinfection efficacy. By leveraging these advanced technologies, WWTPs can achieve more effective and sustainable wastewater treatment, ultimately protecting public health and the environment from the growing threat of antimicrobial resistance [36,47,55].

## 6. Understanding Nanomaterial Photocatalysis: Basics and Mechanisms

Photocatalysis, a process that utilizes light energy to drive chemical reactions, has gained significant attention as a sustainable and effective method for environmental remediation, particularly in the degradation of organic pollutants and disinfection of water [56,57]. The basic principle of photocatalysis involves the absorption of photons by a photocatalyst material, typically a semiconductor, which generates electron–hole pairs. These electron–hole pairs can then participate in redox reactions on the surface of the photocatalyst, leading to the formation of ROS such as hydroxyl radicals (·OH), superoxide anions (O_2_^−^), and singlet oxygen (^1^O_2_), which are potent oxidizing agents capable of breaking down complex organic molecules and inactivating microorganisms, including MDR bacteria [58,59]. The mechanism of photocatalysis begins when the semiconductor material absorbs light energy greater than or equal to its bandgap energy, resulting in the excitation of electrons from the valence band to the conduction band, leaving behind holes in the valence band [60]. The generated electron–hole pairs (e^−^/h^+^) migrate to the surface of the semiconductor, where they can either recombine or interact with adsorbed species, such as oxygen or water, to form ROS. The efficiency of photocatalysis is greatly influenced by the semiconductor’s properties, such as bandgap energy, surface area, and the ability to reduce electron–hole recombination [61,62,63]. Recent advancements in nanotechnology have significantly enhanced the performance of photocatalysts. Nanomaterials, due to their high surface area-to-volume ratio and tunable electronic properties, have been engineered to optimize light absorption and charge separation, thereby improving photocatalytic efficiency [64]. For example, the advancement of metal oxide nanostructures, including titanium dioxide (TiO_2_), zinc oxide (ZnO), cadmium sulfide (CdS), and hybrid nanocomposites, has demonstrated significant photocatalytic activity when subjected to ultraviolet (UV) and visible light irradiation [65,66,67]. Moreover, the incorporation of noble metals (e.g., gold, silver) and non-metal dopants (e.g., nitrogen, carbon) into semiconductor nanomaterials has further enhanced their photocatalytic properties by increasing light absorption in the visible spectrum and reducing charge recombination [68,69].

TiO_2_ in particular has emerged as a promising photocatalyst nanomaterial due to its narrow bandgap (~2.4 eV), which allows it to harness visible light effectively [70]. The generation of ROS under visible light irradiation makes nanomaterials a potent agent for the degradation of organic pollutants and the disinfection of wastewater containing MDR bacteria. The influence of various morphologies on the generation of photogenerated electron–hole pairs was investigated, revealing that some nanosheets exhibited the highest efficiency among the tested structures [71]. The hybrid nanocomposites and nanomaterials, produced using diverse synthesis methods including environmentally friendly biogenic approaches, demonstrate considerable potential in biomedical applications such as antibacterial activity, anticancer treatment, biosensing, and bioimaging. This efficacy can be attributed to their distinctive size, morphology, and biocompatibility [72]. However, the rapid recombination of electron–hole pairs in nanomaterials poses a significant challenge, which has been addressed through the development of nanomaterials-based heterojunctions and composites with other materials that facilitate charge separation and prolong the lifetime of charge carriers [73]. The coupling of photocatalysis with other AOPs, such as ultrasonication and Fenton-like reactions, has been shown to synergistically enhance the generation of ROS, leading to more efficient degradation of pollutants and inactivation of resistant microorganisms [34,74]. Ultrasonication, for example, can create localized high-temperature and high-pressure conditions, known as “hot spots”, that can accelerate chemical reactions and generate additional ROS, thereby complementing the photocatalytic process [75]. The nanomaterial-driven photocatalysis represents a powerful tool for addressing the challenges posed by MDR bacteria and ARGs in wastewater. The continued development of advanced photocatalysts with optimized properties, along with the integration of photocatalysis with other treatment technologies, holds great promise for revolutionizing wastewater treatment and mitigating the global threat of antimicrobial resistance.

### Hybrid Nanocomposites and Nanomaterials in Photocatalytic Disinfection

Hybrid nanocomposites and nanomaterials are emerging as key players in the field of photocatalysis due to their unique optical and electronic properties, which make them highly effective for various environmental applications, including pollutant degradation and antimicrobial activity [76,77]. A II-VI semiconductor has garnered significant interest because of its ability to absorb visible light and generate reactive species that facilitate photocatalytic reactions [78].

Optical Properties: Hybrid nanocomposites and nanomaterials possess a direct bandgap structure, typically measuring approximately 2.4 eV, which enables efficient absorption of visible light. The shape and size of hybrid nanocomposites and nanomaterials can be precisely regulated during the synthesis process, resulting in tunable optical properties [79]. This bandgap energy is critical as it defines the energy necessary for electronic transitions from the valence band to the conduction band, a process that produces electron–hole pairs essential for photocatalytic activity [80]. The photocatalyst demonstrated a 95.6% degradation of tetracycline when exposed to blue light at an intensity of 200 W/m^2^. This size-dependent optical behavior leads to variations in light absorption and emission spectra, thereby enhancing photocatalytic performance under varying light conditions [80,81].

Electronic Properties: The electronic properties of nanomaterials are significantly influenced by their size and morphology. Nanomaterials with high aspect ratios exhibit improved charge carrier mobility and extended excitation lifetimes compared to spherical nanoparticles. When nanomaterials were utilized as an underlayer with a thickness of 40 nm, they significantly reduced the recombination of photoinduced charge carriers, resulting in a 52% enhancement in the photovoltaic performance of solar cells [82]. Additionally, the electron mobility of titanium dioxide (TiO_2_) increased from 3.00 × 10⁻⁵ to 4.13 × 10⁻⁵ cm^2^ V⁻^1^ s⁻^1^ following the incorporation of carbon dots (CDs) [83]. These properties are attributed to reduced charge recombination rates and enhanced surface area-to-volume ratios, which facilitate more efficient charge transfer processes [84]. Furthermore, the one-dimensional structure of nanorods allows for better alignment of electronic bands, which can improve the efficiency of photocatalytic reactions [85].

Surface Properties: The surface characteristics of nanomaterials play a crucial role in their photocatalytic activity. The high surface area of nanorods provides more active sites for adsorption of reactants and subsequent photocatalytic reactions [78,81]. The surface of nanomaterials can be functionalized or modified to enhance their interaction with target molecules or improve their stability and recyclability. For example, doping with additional elements or coupling with various semiconductors can significantly enhance photocatalytic efficiency by altering the electronic structure and improving charge separation [86]. Zinc tin oxide (ZnSnO₃), when combined with MXene—known for its excellent electron transfer properties—exhibits a compatible energy band alignment with nanomaterials, thereby significantly enhancing electron transport and reducing the recombination of photogenerated electron–hole pairs [87]. Moreover, optimizing the ratio of phosphorus doping in nanoparticles has been shown to decrease charge recombination, resulting in increased photocatalytic activity and improved stability in oxidative environments for the degradation of tetracycline antibiotics under blue LED light irradiation [81].

Photocatalytic Mechanism: The photocatalytic mechanism of nanomaterials involves the absorption of photons, which excites electrons from the valence band to the conduction band, creating electron–hole pairs [88]. These charge carriers migrate to the surface of the nanorods, where they can participate in redox reactions. The electrons in the conduction band can reduce molecular oxygen to superoxide radicals (O2^·−^), while the holes in the valence band can oxidize water or organic contaminants to hydroxyl radicals (·OH). The generation of these ROS is essential for the degradation of pollutants and the inactivation of microorganisms [89,90]. The findings indicated that the nanocomposite exhibited significant photocatalytic efficacy in the degradation of malachite green dye. A nanocomposite CdS/ZnIn2S4/graphene was synthesized which resulted in the degradation of malachite green dye within 10 min. The photogenerated holes produced by CdS were transferred to ZnIn_2_S_4_. A fraction of the photogenerated holes directly oxidized the dye adsorbed on the surface of the CdS/ZnIn2S4/graphene nanostructure, while another fraction oxidized H_2_O and –OH to form ^•^OH, which subsequently degraded the dye [91]. The role of photogenerated holes in the degradation process is also prominent. In the case of photoconversion efficiency in dye, photodegradation was notably enhanced at a CdSnO_3_ concentration of 2.5%, attributed to the rapid separation of charge carriers and the plentiful situated at the periphery of nanomaterials [92]. Another ROS that plays a prominent role in the degradation of pollutants and disinfection of pathogens is O_2_**^·−^**. During the photocatalytic process, O_2_**^·−^** is created by the reduction of dissolved oxygen on the surface of the nanomaterials photocatalyst by the acceptance of photogenerated electrons [92].

**Figure 3:**
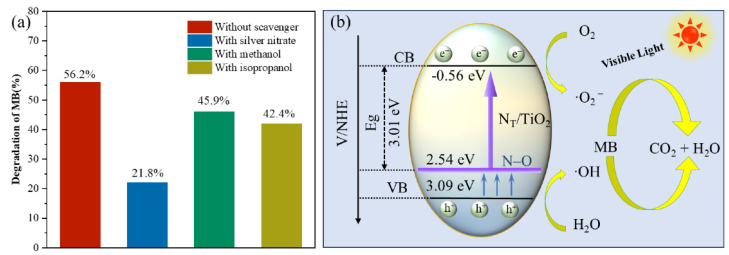
(**a**) Impact of radical scavengers on the photodegradation of methylene blue (MB) and (**b**) a schematic representation illustrating the photocatalytic mechanism of the NT/TiO_2_ photocatalyst. Reprinted with permission from Ref. [102], CC by 4.0.

Application: Stability and Reusability: Hybrid nanocomposites and nanomaterials have demonstrated excellent performance in a range of photocatalytic applications. They have been used effectively for the degradation of organic pollutants, such as dyes and pharmaceuticals, and for the disinfection of water contaminated with pathogenic microorganisms [93,94,95]. The ability of nanomaterials to operate under visible light makes them particularly valuable for practical applications, where solar energy can be harnessed to drive photocatalytic processes. Graphene oxide (GO)-CdS composites, synthesized via a two-phase mixing method, exhibit superior photocatalytic degradation and disinfection efficiencies under visible light, due to enhanced charge transfer, reduced nanomaterials photo-corrosion, and strong GO-CdS interactions [96]. A study suggested the use of bare nanoparticles with high stability of around 30 days to disinfect *E. coli* and *S. aureus* [97]. An efficient photocatalytic ternary nanocomposite g-C_3_N_4_/Cu@CdS was synthesized for efficient degradation of emerging environmental pollutants like ciprofloxacin, dyes, and Gram-positive and -negative bacteria [94]. Photocatalytic activity of rGO/CdS quantum dots was highly mediated by ROS species generation for wastewater disinfection of pathogenic *E. coli* and *S. aureus* [98]. Ternary CdSe Quantum dots/graphene/TiO_2_ composites were synthesized that exhibited superior photocatalytic disinfection activity under visible light to inactivate common coliform indicator organism *E. coli* [99]. One of the challenges with nanomaterials is their stability under prolonged exposure to light and harsh environmental conditions. Some nanomaterials are prone to photocorrosion, which can lead to the dissolution of the material and reduced photocatalytic activity. To address this issue, various strategies have been employed, such as coating nanomaterials with protective layers or integrating them into composite materials to enhance their stability and reusability [100]. For example, cadmium nanomaterials can be combined with other materials such as titanium dioxide (TiO_2_) or carbon-based materials to create heterojunctions that improve charge separation and reduce photocorrosion [101,102]. The properties of nanomaterials, including their optical, electronic, and surface characteristics, make them highly effective photocatalysts. Their ability to absorb visible light, coupled with their high surface area and tunable properties, enhances their performance in photocatalytic applications. Advances in stabilizing and optimizing nanomaterials continue to expand their potential for addressing environmental challenges, particularly in wastewater treatment and antimicrobial applications. Figure 3 presents important insights into the role of radical scavengers in the photodegradation process of methylene blue (MB) and the underlying photocatalytic mechanism of the NT/TiO_2_ photocatalyst. The data shown in part (a) indicate that the presence of radical scavengers significantly influences the degradation efficiency of MB, suggesting that reactive species play a critical role in the photocatalytic process. This highlights the importance of identifying and managing radical species to optimize photocatalytic activity. In part (b), the schematic diagram illustrates the photocatalytic mechanism, demonstrating how the NT/TiO_2_ photocatalyst generates reactive oxygen species (ROS) under light irradiation, which subsequently facilitates the degradation of MB. Together, these components underscore the complexity of photocatalytic systems and the necessity of understanding radical dynamics to enhance the efficiency of wastewater treatment processes. When a photocatalytic reaction involves two different semiconductors along with an electron donor–acceptor pair, a Z-scheme mechanism occurs. The Z-scheme photocatalytic mechanism illustrates the transfer of electrons and holes across two photocatalysts coupled together, mimicking the photosynthesis electron flow in nature. In this system, two different photocatalysts are combined, each absorbing light and generating electron–hole pairs. The higher-energy electrons in the conduction band (CB) of the first photocatalyst transfer to the lower-energy conduction band of the second photocatalyst, while holes move in the opposite direction. This separation of charge carriers reduces recombination and allows for a more effective generation of ROS, such as hydroxyl and superoxide radicals, essential for disinfection. The Z-scheme facilitates efficient radical production and enhances overall photocatalytic efficiency by optimizing the energy bands, significantly improving disinfection applications.

## 7. Visible Light Photocatalysis via LED Irradiation

The incorporation of LED irradiation in photocatalysis has emerged as a transformative approach, significantly improving the efficiency of photocatalytic processes, especially when used in conjunction with nanomaterials such as Bi/Bi_3_NbO_7_, PbS/CaCO_3_, AgBr/CuBi_2_O_4_ composite, magnetic ultrathin γ-Fe_2_O_3_ nanosheets/mesoporous black TiO_2_ hollow sphere (γ-Fe_2_O_3_/b-TiO_2_), etc. [103]. LED light is particularly advantageous due to its specific wavelength output, which can be tailored to match the absorption spectra of photocatalysts, thereby maximizing their activation. This enhancement facilitates the generation of reactive oxygen species (ROS) crucial for the degradation of a wide array of environmental contaminants, including organic pollutants and pathogenic microorganisms. The synthesis of those composites nanoparticles through techniques like hydrothermal, solvothermal, sonochemical, and chemical bath deposition allows for precise control over their physicochemical properties, such as particle size, crystallinity, and surface area, which are critical for optimizing photocatalytic performance [103]. Advanced modifications, including metal doping and hybridization with carbon-based materials like graphene, have shown to not only improve the electronic properties and charge carrier dynamics of nanocomposites but also enhance their structural stability under operational conditions. These modifications lead to increased light absorption, reduced electron–hole recombination, and enhanced photocatalytic activity, allowing for effective degradation of challenging pollutants such as methyl orange, rhodamine B, and a range of antibiotics [103]. As a result, hybrid nanomaterial-based photocatalysts represent a promising and sustainable solution for addressing the pressing issue of wastewater contamination, particularly in the context of emerging pollutants that pose risks to environmental and human health.

**Figure 4:**
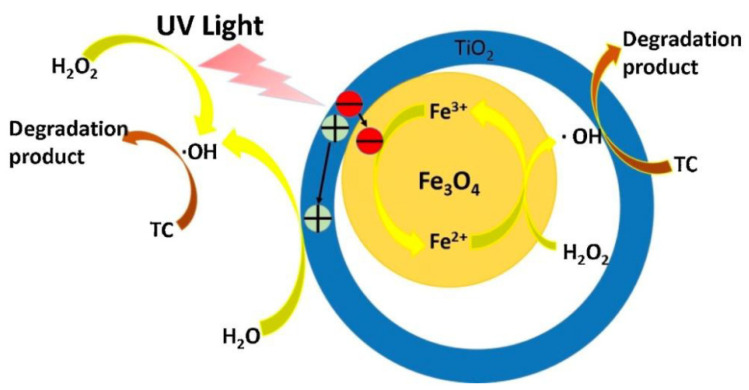
Mechanism of tetracycline degradation via photo-Fenton-like reaction. Reprinted with permission from Ref. [103].

Figure 4 illustrates the mechanism involved in the degradation of tetracycline through a photo-Fenton-like reaction. This process utilizes hydroxyl radicals (•OH), which are generated when iron ions (Fe^2^⁺) react with hydrogen peroxide (H_2_O_2_) under UV or visible light irradiation. These highly reactive radicals play a crucial role in breaking down the tetracycline molecules into less harmful byproducts. The photo-Fenton-like system accelerates the production of reactive oxygen species (ROS), enhancing the oxidative degradation of tetracycline. This figure highlights how iron catalysis, coupled with light exposure, enhances the efficiency of the Fenton reaction, making it an effective approach for removing persistent organic pollutants like tetracycline from water.

### 7.1. Benefits of LEDs Compared to Conventional Light Sources

Blue LED irradiation has emerged as a highly effective alternative to traditional light sources in photocatalysis, offering notable advantages in efficiency, cost-effectiveness, and environmental sustainability. One of the key benefits of blue LEDs is their exceptional energy efficiency, as they convert a significantly higher percentage of electrical energy into visible light compared to conventional sources. For instance, incandescent bulbs typically convert only about 5–10% of their electrical energy into visible light, with the majority (90–95%) being dissipated as waste heat, primarily in the infrared spectrum [103,104]. In contrast, blue LEDs achieve an efficiency of approximately 40–50%, substantially reducing energy consumption and associated operational costs while promoting sustainability. Additionally, blue LEDs possess considerably longer lifespans, ranging from 25,000 to 50,000 h of continuous operation, which far exceeds the longevity of ultraviolet (UV) lamps and tungsten bulbs [105]. This durability minimizes the need for frequent replacements and lowers maintenance costs, contributing to long-term financial savings. Furthermore, blue LEDs generate significantly less heat than traditional light sources, thereby preserving the stability of photocatalysts and the treated solutions, ultimately enhancing the efficacy of photocatalytic reactions. Environmentally, blue LEDs are regarded as a greener option since they do not contain harmful substances such as mercury, which is commonly found in UV lamps and poses risks of environmental contamination. Their energy efficiency and longevity also translate into a reduced carbon footprint due to lower energy requirements and decreased waste generation compared to conventional lighting solutions. Despite the higher upfront investment, the long-term benefits of blue LEDs—such as reduced operational and maintenance costs coupled with improved photocatalytic performance—render them a financially and environmentally viable option for advancing photocatalytic technologies.

### 7.2. Blue LEDs in Boosting Photocatalytic Performance

Blue LEDs typically emit light in the wavelength range of 450–495 nm, which corresponds to the range of wavelengths that are effectively absorbed by certain photocatalysts, including hybrid nanocomposites and nanomaterials [80,103,106]. The energy from blue LED light is absorbed by the photocatalyst, exciting electrons from the valence band to the conduction band, thereby generating electron–hole pairs [107]. These electron–hole pairs are crucial for the formation ROS which are responsible for the oxidative degradation of organic pollutants and the disinfection of microorganisms [108].

The energy provided by blue LED light is particularly effective in exciting hybrid nanocomposites and nanomaterials due to their band gap energy, which aligns well with the photon energy of blue light [109]. This alignment enhances the efficiency of the photocatalytic process by promoting the generation of ROS and improving the overall rate of photocatalytic reactions. Additionally, blue light has been shown to minimize the formation of unwanted by-products and reduce the recombination rate of electron–hole pairs, thereby increasing the longevity and efficacy of the photocatalytic reaction [80,81]. Research has demonstrated that blue LED irradiation significantly enhances the photocatalytic activity of hybrid nanocomposites and nanomaterials. For instance, studies have reported that the degradation rate of organic pollutants, such as dyes and pharmaceuticals, is markedly improved under blue LED light compared to other light sources. A study with zinc porphyrin has shown Blue LED enhances the photocatalytic activity by providing visible-light irradiation that effectively activates the graphene quantum dots and zinc porphyrin nanocomposite for the degradation of organic pollutants like methylene blue [110]. The efficiency of this photocatalytic process is influenced by factors such as light intensity and the physical properties of the hybrid nanocomposites and nanomaterials, including their size and morphology. Furthermore, the schematic emphasizes the potential for hybrid nanocomposites and nanomaterials to act synergistically with other materials or methods, thereby enhancing the overall degradation efficiency. This understanding of the underlying photocatalytic mechanisms is essential for optimizing the use of hybrid nanocomposites and nanomaterials in wastewater treatment applications, particularly in the context of addressing persistent organic pollutants and combating antimicrobial resistance. Furthermore, the use of blue LEDs has been associated with improved disinfection efficiency. Blue light-induced photocatalysis has been effective in inactivating a broad spectrum of microorganisms, including bacteria, viruses, and fungi [73,111]. The ROS generated under blue LED light are highly reactive and capable of penetrating microbial cell membranes, leading to cell damage and inactivation [112]. This characteristic makes blue LED-activated photocatalysis a promising approach for addressing microbial contamination in wastewater and other environmental matrices.

### 7.3. Optimization of Blue LED Irradiation Parameters

To maximize the effectiveness of blue LED irradiation in photocatalytic processes, several parameters need to be optimized, including light intensity, irradiation time, and the distance between the LED source and the photocatalyst. Higher light intensity generally leads to increased production of ROS and enhanced photocatalytic activity [113,114]. However, excessive light intensity can also cause overheating and degradation of the photocatalyst, which must be carefully managed [115]. The irradiation time is another critical factor that influences photocatalytic performance. Prolonged exposure to blue LED light typically results in more extensive degradation of pollutants and more effective microbial inactivation [116]. However, the balance between effective treatment and energy consumption should be considered to ensure the sustainability of the process [117].

## 8. Hybrid Photocatalytic Disinfection System

A hybrid energy system often comprises of two or more energy sources or methods that are utilized in conjunction with appropriate energy conversion techniques to provide fuel savings, energy recovery, and enhanced efficiency. The integration of various disinfection systems into a singular hybrid, such as those involving physical, biological or chemical systems, leverages its properties to enhance overall treatment efficiency. The synergistic interaction between the processes results in a more robust and effective disinfection process. This hybrid approach can overcome some of the limitations of traditional disinfection methods, offering a more efficient solution for managing wastewater and combating resistant microorganisms.

### 8.1. Integration of Ultrasonication to Enhance Disinfection

Ultrasonication, the use of high-frequency sound waves on a liquid medium, is a potential method for improving disinfection processes, especially in wastewater treatment. Acoustic cavitation is the main process by which ultrasonication causes disruption of bacterial cells. This process produces powerful physical forces that may efficiently harm and eliminate microorganisms, even germs that are resistant to many drugs [118].

Acoustic cavitation refers to the formation, growth, and implosion of microscopic bubbles in a liquid medium due to the application of sound waves [119]. This phenomenon may be used to disrupt cells, causing their structural integrity to be compromised. The principle of acoustic cavitation states that ultrasonication works by generating ultrasonic waves, usually within the frequency range of 20 kHz to 1 MHz, into a liquid. Acoustic cavitation is the phrase used to describe the development and collapse of small gas bubbles in a liquid, caused by waves that generate alternating high- and low-pressure areas. The fast disintegration of these bubbles creates concentrated elevated temperatures and pressures, resulting in shock waves and microjets that have the potential to cause substantial physical damage to bacterial cells. The mechanical forces caused by cavitation, such as shock waves and microjets, may disrupt the cell membranes of bacteria. The pressures mentioned cause the creation of holes or total rupture of the cell membranes, leading to the release of intracellular contents and ultimately causing the death of the cell [120]. The efficacy of this procedure is affected by several aspects, such as ultrasonic frequency, power intensity, and exposure duration. The damage caused to the cell membrane hinders the ability of bacteria to live and multiply [75]. Ultrasonication not only causes physical disturbance, but also increases the production of ROS in the liquid media. Cavitation generates high-energy conditions that facilitate the production of ROS, including hydroxyl radicals. These ROS play a crucial role in eliminating bacteria by targeting biological components such as proteins, lipids, and nucleic acids. The combination of mechanical disruption and chemical oxidation significantly enhances the efficacy of ultrasonication in disinfection procedures [121,122,123].

Ultrasonication has demonstrated to have synergistic effects when used in conjunction with other disinfection approaches, such as photocatalysis or chemical disinfectants. For instance, Yentür et al. [124] and Rahman et al. [125] showed that the combination of ultrasonication and photocatalytic processes greatly improved the elimination and oxidation of pharmaceutical drugs and of MDR bacteria from wastewater by utilizing both mechanical and oxidative mechanisms [124,125]. This integrated strategy enhances the overall effectiveness of disinfection and decreases the amount of necessary chemical agents. The ability to scale-up ultrasonication for wastewater treatment has been extensively studied. Numerous research studies have concentrated on improving operating parameters and evaluating the economic viability of applying this technology on a wider scale. Several setups of ultrasonication systems were studied, and it was determined that controlling variables such as ultrasonic power and treatment duration might enhance the cost-effectiveness of this technology in industrial settings [126]. Table 1 presents a comprehensive compilation of research focused on the application of ultrasonication as a method for controlling pathogenic bacteria. Ultrasonication, which utilizes high-frequency sound waves, has emerged as a promising technique for enhancing the inactivation of harmful microorganisms.

The table highlights various studies that detail the specific conditions, such as frequency and duration of ultrasonication, as well as the bacterial strains targeted. By summarizing the findings from these studies, the table provides insights into the effectiveness of ultrasonication, contributing to the understanding of its potential as an alternative or complementary disinfection strategy [34,127,128,129,130,131,132,133,134,135,136,137]. This compilation could serve as a valuable resource for researchers and practitioners aiming to explore innovative approaches to microbial control in various settings, including food safety and water treatment.

### 8.2. Enhancing Disinfection with Plant Active Compounds

The use of plant active products as natural biocides, such as terpenes, in wastewater disinfection has become a viable and long-term solution to the problem of bacteria that are resistant to drugs [138]. Plant-derived chemical molecules called terpenes have been explored extensively for their potential applications in medicine and food preservation due to their broad-spectrum antibacterial capabilities [139]. The use of terpenes in wastewater treatment provides a natural, biodegradable substitute for traditional chemical disinfectants, which can have negative effects on the environment and can promote the growth of microbial resistance [140,141].

The monoterpene terpinolene, which is frequently present in plants such as citrus fruits, tea trees, and conifers, has strong antibacterial properties against a variety of diseases, including MDR species like *Klebsiella pneumoniae*, *Staphylococcus aureus*, and *Escherichia coli* [140,142]. These pathogens are well-known for their resilience to conventional disinfection procedures and their role in escalating antibiotic resistance within the ecosystem. The bacterial cell membrane of the bacterium is disrupted by terpinolene, which causes leaking of cellular contents and ultimately results in cell death [143]. The compound’s lipophilic properties, which enable it to integrate into and destabilize the lipid bilayer of bacterial membranes, are principally responsible for this disruption [144].

Recent research has demonstrated that terpinolene and ultrasonication, a technique that employs high-frequency sound waves to produce acoustic cavitation, work particularly well together to improve wastewater disinfection [30,145,146]. Bacterial cells are physically disrupted by the strong physical pressures produced by acoustic cavitation, such as shock waves and microjets. The mechanical and chemical actions of terpinolene combine to improve the effectiveness of bacterial inactivation in this system. As a consequence, compared to conventional approaches, the disinfection process is more efficient and can considerably lower the bacterial burden in a shorter amount of time [118,147]. The application of using natural biocide like terpenes in wastewater treatment has significant practical ramifications, such as the drawbacks of chemical disinfectants, such as how they can produce toxic by-products like chlorophenols that can be much more dangerous to human health. Additionally, terpinolene is a naturally occurring substance that decomposes biologically, lessening the disinfection process’s environmental impact. Studies have shown that it is stable during the disinfection process, indicating that it may be recovered and reused. This makes it an economical choice as well. Large-scale operations benefit greatly from this reusability because ongoing chemical input costs might be prohibitive [148].

## 9. Lowering Antibiotic Resistance Genes

ARGs are a major problem in wastewater treatment because of their persistence and ability to transmit antibiotic resistance. Enhancing wastewater treatment procedures and reducing hazards to the public’s health require efficient methods to break down or eliminate ARGs. ARGs are reduced by a number of processes, chief among which is the production of ROS and other substances that interfere with and break down these resistant genetic components. ARGs pose a significant challenge in wastewater treatment due to their persistence and potential to contribute to the spread of antimicrobial resistance. Effective strategies to degrade or remove ARGs are crucial for improving wastewater treatment processes and mitigating public health risks. The reduction in ARGs involves various mechanisms, primarily the generation of ROS and other factors that disrupt and degrade these resistant genetic elements. The most commonly detected antibiotics and their related ARGs include sulfonamides (e.g., *Sul1* and *Sul2*), fluoroquinolones (e.g., *qnrA*, *qnrB*, and *qnrC*), tetracyclines (e.g., *tetA*, *tetB*, *tetC*, and *tetW*), and β-lactam (e.g., *bla_TEM_* and *bla_DHA_*) [149,150]. In general, ARGs are present in the form of intracellular ARGs and extracellular ARGs. Notably, external ARGs are generated by the release of internal secretion from bacterial cells having a shorter half-life and less abundance. In addition, ARGs can also be free and adsorbed, where they are adsorbed onto cells and other particles. It may also be noted that different ARGs have different reactivity towards oxidative processes. Like chlorination, disinfection can reduce some ARGs like *tetM* and *gyrA* but causes an increase in *vanA*. [151]. ROS are highly reactive molecules that can damage cellular components, including nucleic acids, proteins, and lipids. ROS play a critical role in the degradation of ARGs through oxidative damage to DNA where direct ROS attack such as by hydroxyl radicals (•OH) can induce oxidative damage to DNA by causing strand breaks and modifying nucleotides [152]. This damage can lead to the loss or alteration of genetic information, including ARGs. For instance, hydroxyl radicals can initiate oxidative cleavage of DNA strands, disrupting the integrity of ARGs and reducing their potential for horizontal gene transfer [152]. ROS can also form adducts with DNA bases, leading to mutations and structural changes that hinder the expression and replication of ARGs. These modifications can reduce the functionality of ARGs and decrease their prevalence in the microbial community [153].

## 10. Improved ROS Generation for ARB and ARG Removal

Enhanced ROS generation in hybrid photocatalytic disinfection systems is a critical advancement in the field of wastewater treatment, offering a more effective approach to inactivating pathogens and degrading ARGs. Hybrid systems combine traditional photocatalysis with other complementary techniques, such as ultrasonication, ozonation, or Fenton-like reactions, to amplify the production of ROS like hydroxyl radicals (·OH), superoxide anions (O_2_·⁻), and hydrogen peroxide (H_2_O_2_). The integration of photocatalysts such as CdS nanorods in wastewater treatment systems enhances ROS generation under light irradiation. This increased ROS production can improve the efficiency of ARG degradation by promoting more extensive oxidative damage. Studies have shown that photocatalytic systems with optimized light intensity and catalyst dosage can significantly enhance ROS levels and accelerate ARG removal. Combining photocatalysis with other methods, such as ultrasonication or natural biocides, can further boost ROS generation and improve ARG degradation. For example, the combined use of CdS nanorods and terpinolene can lead to synergistic effects, enhancing the overall disinfection efficiency and ARG reduction. The effects of ultrasonication causes physical disruption by generating acoustic cavitation, which produces high-energy shock waves and microbubbles. Table 2 provides a comprehensive overview of research focused on the disinfection of pathogenic bacteria through the combined application of ultrasonication and photocatalysis. This table summarizes various studies that explore the effectiveness of different ultrasonication parameters, such as frequency and duration, alongside specific photocatalysts utilized in the disinfection process. The findings presented demonstrate the varying levels of disinfection efficacy achieved against several pathogenic bacteria, highlighting the potential of these hybrid methods in microbial control. By integrating ultrasonication with photocatalysis, researchers aim to enhance the inactivation rates of bacteria, offering insights into more effective wastewater treatment solutions. This compilation serves as a valuable resource for further investigation into the synergistic effects of these techniques in pathogen management.

## 11. Eco-Friendly Hybrid Disinfection Solutions

The long-term efficacy and sustainability of nanomaterials are crucial for their application in wastewater treatment. Understanding the durability and reusability of these nanomaterials is essential for evaluating their practical utility in continuous and sustainable treatment processes. Nanomaterials are recognized for their remarkable stability and reusability in photocatalytic applications, making them an effective choice for long-term use in environmental remediation. Under continuous light irradiation, nanomaterials and their composites demonstrate impressive photocatalytic stability, retaining over 90% of their initial activity even after 100 h of exposure to UV or visible light, as highlighted by Yang et al. [159]. This durability is largely due to their robust crystal structure, which resists photodegradation, ensuring consistent performance over extended periods.

In addition to their stability under light, nanomaterials also exhibit significant chemical and mechanical resilience. They are resistant to chemical degradation in various aqueous environments, which is crucial for maintaining high photocatalytic efficiency amidst different water contaminants, including acids and bases [160]. Mechanically, nanomaterials can withstand dynamic treatment processes, such as repeated mechanical agitation and filtration, without losing their catalytic properties. In the hybrid disinfection system, particularly in sonophotocatalysis, the synergy between ultrasonic waves and photocatalysis significantly enhances the degradation of pollutants and pathogens. This synergy is quantified using the synergy index, which compares the combined effect of ultrasound (US) and ultraviolet (UV) light with their individual effects. The synergy index (SI) can be calculated using the Equation (1) [161]:

(1) $$Synergy\;(\%)=\frac{K_{\left(cat+LED+US\right)}-(K_{\left(cat+LED\right)}+K_{\left(cat+US\right)}\;)}{K_{\left(cat+LED+Us\right)}\;}$$

where *K* represents the various rate of reactions for bacterial log reduction. $K_{\left(cat+LED+US\right)}$ represents the rate of reaction of the hybrid system and $K_{\left(cat+LED\right)}\;$ and $K_{\left(cat+US\right)}$ represent the rate constants of photocatalysis and sonocatalysis alone. The electrical energy (EE) consumption is another critical parameter when evaluating the cost-effectiveness of sonophotocatalytic systems. The EE consumption is calculated to assess the energy efficiency of the process, expressed in kilowatt-hours per cubic meter (kWh/m^3^). This calculation includes the energy consumed by both the ultrasonic generator and the light source. The total EE consumption can be calculated using the below equation [162]:

$$EE=\frac{P_{el}t\;(1000\;)}{V60\log\left(\frac{C_{0}}{C_{t}}\right)}$$

Here, E_0_ is the electrical energy in kW-h required to reduce bacterial concentration by 1 order of magnitude. *P_el_* is the input power of the electrical source in W, V is the reaction volume in L, *t* denotes time in seconds, and *C*_0_ and *C_t_* are initial and final bacterial concentrations, respectively.

The integration of these processes not only enhances disinfection efficiency but also reduces operational costs by optimizing material cost and energy usage. In a study by Mohseni-Bandpei et al. [163], it was described that the necessary quantities of ZnO and a photosensitizer, together with their corresponding prices, for treating 1 m^3^ of wastewater containing 200 mg/L of the photosensitizer were USD 1690.5 and USD 1064, respectively. The combined system (ZnO/photosensitizer/UV/US) had an energy cost of USD 7.1 and material costs of USD 92.4 and USD 55.1 for ZnO and the photosensitizer, respectively. This clearly demonstrates the cost-effectiveness of a hybrid system. Also, the photocatalytic materials used can be recovered and recycled, which further reduces the operational cost. Reactor design and the type of light used can promote a longer operational runtime. As already mentioned, LED lights can replace inefficient traditional lights and can also replace unreliable sunlight, which can affect the efficiency of the photocatalytic process.

## 12. Conclusions and Future Directions

In conclusion, the application of hybrid materials in advanced disinfection techniques represents a promising frontier in wastewater treatment, with potential implications for broad environmental and public health benefits. Hybrid disinfection systems, particularly those integrating photocatalytic nanomaterials with natural biocides or metallic dopants, address critical limitations observed in conventional photocatalysis, including mass transfer restrictions, recombination issues, and catalyst fouling. The synergistic effect of combining different components within hybrid materials allows for the generation of highly reactive oxygen species (ROS), which enhance bacterial inactivation by targeting multiple microbial structures. By improving light absorption and expanding activity to the visible spectrum, these systems increase versatility, enabling effective performance across a range of conditions. Additionally, optimizing operational parameters, such as catalyst concentration and light intensity, further refines disinfection efficiency, achieving rapid microbial inactivation with lower energy requirements. Overall, the research underscores the potential of hybrid systems to deliver more robust, efficient, and rapid disinfection methods suitable for real-world applications. As further studies explore material compatibility, mechanisms of action, and scale-up potential, hybrid-material-based disinfection could play a transformative role in addressing global challenges related to waterborne pathogens and wastewater treatment. However, considerations such as the environmental stability and potential ecotoxicity of nanomaterials within these systems must be addressed to ensure safe, sustainable deployment in large-scale applications. Future research should continue to explore diverse hybrid combinations, optimize design for specific pollutants, and address regulatory challenges to facilitate the practical application of these innovative technologies.

Comprehensive Targeting of ARGs and MDR Bacteria: This review demonstrates that the ROS generated within the system not only inactivate bacteria but also damage nucleic acids, inhibiting horizontal gene transfer (HGT) and the propagation of antimicrobial resistance (AMR). This dual-action approach effectively targets multidrug-resistant (MDR) bacteria and antibiotic resistance genes (ARGs), making it a robust AMR mitigation strategy in wastewater treatment.

Researchers are also working on modifying nanocatalysts, such as by selective metal doping and forming efficient heterojunctions, to further enhance sonophotocatalytic efficiency. These modifications improve light absorption, optimize charge transfer pathways, and minimize electron–hole recombination, leading to more effective ROS production. Incorporating natural biocides like terpenes adds an additional level of effectiveness, as they work synergistically with ultrasonication and photocatalysis to disrupt bacterial membranes and metabolic processes. This combination ensures sustained photocatalytic activity over multiple cycles, making it a viable and long-term solution for wastewater treatment. The integration of acoustic cavitation, photocatalysis, and natural biocides significantly enhances disinfection efficiency while offering an energy-efficient solution. Investigating innovative methods to generate cavitation could further lower treatment costs and enable the integration of sustainable energy sources. However, for large-scale implementation, detailed economic analyses are crucial. These assessments must account not only for energy consumption but also for expenses related to reactors, operational systems, and the procurement of cost-effective natural biocides.

Despite notable advancements, linking nanomaterial properties with sonophotocatalytic performance remains challenging due to variations in contaminants and operating conditions across different studies. Nevertheless, nanostructures such as nanotubes and nanorods consistently demonstrate superior performance, providing greater surface area for enhanced interaction with contaminants and photons. Looking ahead, the future success of sonophotocatalysis will rely on optimizing the degradation of multiple contaminants, standardizing experimental methodologies, and gaining a deeper understanding of the underlying reaction mechanisms. Special attention should focus on the development of Z-scheme sonophotocatalytic systems, which can minimize energy losses in large-scale applications, and evaluating the long-term stability of nanomaterials in practical conditions. Furthermore, thorough economic evaluations will be vital to ensure the scalability, sustainability, and cost-effectiveness of this technology for widespread use, particularly in tackling multidrug-resistant (MDR) bacteria and antibiotic resistance genes (ARGs) in wastewater.

## Figures and Tables

**Table 1:** Summary of studies examining the effects of ultrasonication on pathogenic bacteria.

Pathogenic Bacteria	Ultrasonication Parameters	Disinfection Time (mins)	Findings	Ref.
*Staphylococcus aureus (+)*	26 kHz;	5–30	Prolonged disinfection duration correlates with higher bacterial cell mortality	[127]
*Escherichia coli (−)*	26 kHz;	5–30	E. coli less resistant compared to Gram-positive bacteria	[127]
*Staphylococcus aureus (+)*	20 kHz; 60 W cm^−2^	30	Disinfection efficacy improves with higher ultrasound power	[128]
*Staphylococcus aureus (+)*	20 kHz; Power input power 63% (950 W)	3–15	Damage to membrane coupled with metabolic inhibition	[129]
*Bacillus subtilis (+)*	20 kHz; 20−500 W	30	Reduction in spores accompanied by compromised integrity of the inner membrane	[130]
*Bacillus subtilis (+)*	20 kHz and 250 W and 90 W	5–15	Inhibition of growth in vegetative cells by bacteriostatic agents	[130]
*Escherichia coli (−)*	37 kHz; 280 W	5–11	Hybrid effect of ultrasonic electrocoagulation	[131]
*Escherichia coli (−)*	50 kHz; 120 W	10–20	A 97% reduction in cell viability through combined sonophotocatalysis, resulting in cell death from ROS and membrane depolarization	[132]
*Escherichia coli (−)*	22, 36, 833 kHz; 95 W	10–60	Optimal *E. coli* inactivation achieved with hybrid ultrasound–silver systems	[133]
*Escherichia coli (−)*	20 kHz; input power 100 W; Power intensity 50 and W cm^−2^	7	Cell membrane integrity compromised, resulting in loss of DNA and ATP	[134]
*Pseudomonas aeruginosa (−)*	60, 100 kHz; 15 and 26.32 W/cm^2^;	15	Metabolic activity and osmotic pressure of bacterial biofilm disrupted, leading to inhibition of protein synthesis	[135]
*Pseudomonas aeruginosa (−)*	100 kHz; 25% duty cycle	15–60	Biofilm disruption resulting in over 1-Log CFU reduction	[136]
*Klebsiella pneumonia (−)*	40 kHz; 410–700 W	10–20	Sono-photocatalysis achieved 6-Log CFU disinfection	[34]
*Klebsiella pneumonia (−)*	40 kHz; Intensity 92.36 mW/cm^2^	5	The combination of ultrasound and antibiotics diminished biofilm formation on catheters	[137]

**Table 2:** Pathogenic bacteria disinfection via ultrasonication and photocatalytic methods.

Photocatalyst Used and Light Source, Intensity	Synthesis Method, Morphology, Size	Ultrasonication Parameters	Pathogenic Bacteria and Reaction Time (mins)	Findings	Ref.
ZnO:Fe, Sunlight, 55,000 ± 5000 lux	Chemical precipitation, near-spherical, 80–100 nm	40 kHz bath sonicator	*Salmonella* sp., 120	Complete disinfection for 7-log colonies	[154]
ZnO, Visible LED, 70,000 lux	Precipitation, spherical, 100 nm	40 kHz bath sonicator	*Shigella* sp., 120	Complete disinfection	[125]
CdS nanorods, Blue LED, 15 mW/cm^2^	Hydrothermal, nanorods, Dia = 45–55 nm; Length = 1 nm	40 kHz; 700 W	*Klebsiella pneumonia*, 20	6-log bacterial disinfection	[34]
ZnO nanofluids, Natural light	Commercial ZnO nanopowder, 90–200 nm	20 kHz and 90 W/L	*Escherichia coli*	Max efficiency = US + Light + ZnO	[155]
Ag, UV of 254 nm	Ag + brick dust in filled column	40 kHz	*Escherichia coli*, 25	Synergistic effect in a US/UV mode + Ag	[156]
TiO_2_, UV-C 254 nm, 3.25–3.27 mW/cm^2^	TiO_2_ salt as photocatalyst	24 kHz, 400 W	*Aeromonas hydrophila*, 20	8-log reduction	[157]
CdS nanorods + Terpinolene, Blue LED, 15 mW/cm^2^	Hydrothermal, nanorods, Dia = 45–55 nm; Length = 1 nm	40 kHz, 700 W	Modification of secondary wastewater effluent using *Enterobacter* sp., *Escherichia coli*, and *Klebsiella pneumonia*, 10	Synergistic effect of photocatalyst + US + biocide in complete disinfection	[30]
BaZr_0_._02_Ti_0_._98_O_3_, UV 365 nm,	Solid-state reaction, random morphology, 0.1–3 µm	40 kHZ, 70 W	*Escherichia coli*, 120	80% disinfection	[158]
CdS, Blue LED, 15 mW/cm^2^	Hydrothermal, nanorods, Dia = 45–55 nm; Length = 1 nm	40 kHz, 700 W	*Klebsiella pneumonia*, 20	MDR bacteria, 6-log disinfection (100%)	[34]
